# Tsunami Runup and Inundation in Tonga from the January 2022 Eruption of Hunga Volcano

**DOI:** 10.1007/s00024-022-03215-5

**Published:** 2022-12-28

**Authors:** Jose C. Borrero, Shane J. Cronin, Folauhola Helina Latu’ila, Pupunu Tukuafu, Nikolasi Heni, Ana Maea Tupou, Taaniela Kula, Ofa Fa’anunu, Cyprien Bosserelle, Emily Lane, Patrick Lynett, Laura Kong

**Affiliations:** 1 eCoast Marine Consulting and Research, Raglan, New Zealand; 2 Tsunami Research Center, Sonny Astani Department of Civil and Environmental Engineering, University of Southern California, Los Angeles, CA USA; 3 Department of Geology and Earth Science, University of Auckland, Auckland, New Zealand; 4 Tonga Geological Services, Nuku’alofa, Tonga; 5 Tonga Meteorological Service, Nuku’alofa, Tonga; 6 National Institute of Water and Atmospheric Research, Taihoro Nukurangi, Christchurch, New Zealand; 7 UNESCO/IOC International Tsunami Information Centre, Honolulu, HI USA

**Keywords:** Tsunamis, volcanic hazard and risk, field survey, eyewitness

## Abstract

On January 15th, 2022, at approximately 4:47 pm local time (0347 UTC), several weeks of heightened activity at the Hunga volcano 65 km northwest of Tongatapu, culminated in an 11-h long violent eruption which generated a significant near-field tsunami. Although the Kingdom of Tonga lies astride a large and tsunamigenic subduction zone, it has relatively few records of significant tsunami. Assessment activities took place both remotely and locally. Between March and June 2022, a field team quantified tsunami runup and inundation on the main populated islands Tongatapu and Eua, along with several smaller islands to the north, including the Ha’apai Group. Peak tsunami heights were ~ 19 m in western Tongatapu, ~ 20 m on south-eastern Nomuka Iki island and ~ 20 m on southern Tofua, located ~ 65 km S and E and 90 km N from Hunga volcano, respectively. In western Tongatapu, the largest tsunami surge overtopped a 13–15 m-high ridge along the narrow Hihifo peninsula in several locations. Analysis of tide gauge records from Nukualofa (which lag western Tongatapu arrivals by ~ 18–20 min), suggest that initial tsunami surges were generated prior to the largest volcanic explosions at ~ 0415 UTC. Further waves were generated by ~ 0426 UTC explosions that were accompanied by air-pressure waves. Efforts to model this event are unable to reproduce the timing of the large tsunami wave that toppled a weather station and communication tower on a 13 m-high ridge on western Tongatapu after 0500 UTC. Smaller tsunami waves continued until ~ 0900, coincident with a second energetic phase of eruption, and noted by eyewitnesses on Tungua and Mango Islands. Despite an extreme level of destruction caused by this tsunami, the death toll was extraordinarily low (4 victims). Interviews with witnesses and analysis of videos posted on social media suggest that this can be attributed to the arrival of smaller ‘pre tsunami’ waves that prompted evacuations, heightened tsunami awareness due to tsunami activity and advisories on the day before, the absence of tourists and ongoing tsunami education efforts since the 2009 Niuatoputapu, Tonga tsunami. This event highlights an unexpectedly great hazard from volcanic tsunami worldwide, which in Tonga’s case overprints an already extreme level of tectonic tsunami hazard. Education and outreach efforts should continue to emphasize the ‘natural warning signs’ of strong ground shaking and unusual wave and current action, and the importance of self-evacuation from coastal areas of low-lying islands. The stories of survival from this event can be used as global best practice for personal survival strategies from future tsunami.

**Supplementary Information:**

The online version contains supplementary material available at 10.1007/s00024-022-03215-5.

## Introduction

**Fig. 1:**
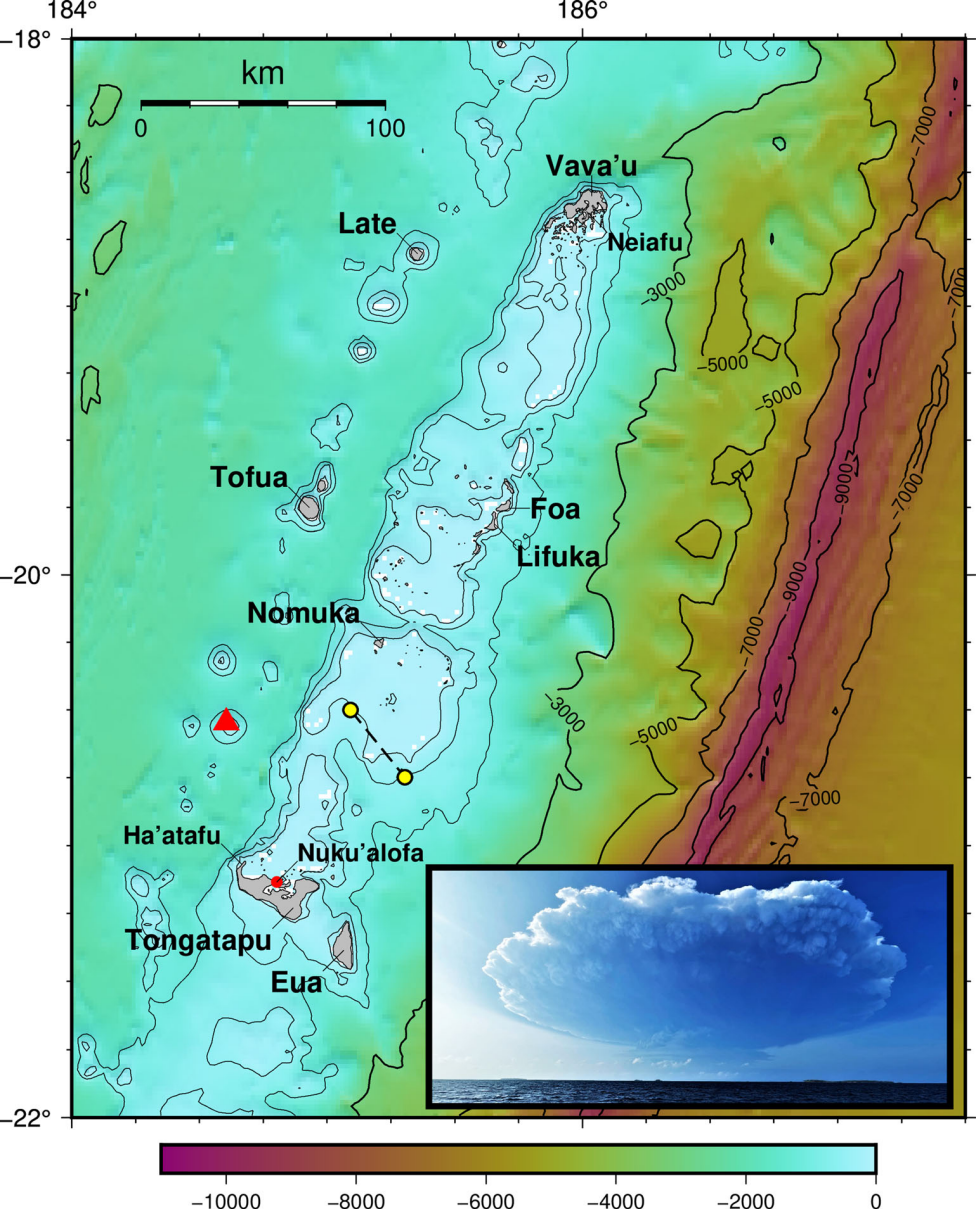
The bathymetry surrounding the major islands of The Kingdom of Tonga. Red triangle shows the location of Hunga Volcano. Yellow dots are the location of the Tongan Navy vessel on January 15th. Inset shows the eruption rising to its climax at 5.24 pm (0424 UTC) January 15th as seen from ~ 5 km northeast of Nuku’alofa (Photo: Branko Sugar); the upper plume is already > 100 km wide

On January 15th, 2022, at approximately 4:47 pm local time (0347 UTC), several weeks of heightened activity at Hunga volcano culminated in an 11-h long violent eruption, 65 km northwest of the main populated island of Tongatapu in the Kingdom of Tonga (Fig. 1). Hunga is often referred to by the names of two small islands Hunga-Tonga and Hunga Ha’apai that are located on the submarine caldera’s northern rim (Cronin et al., 2017), During the first 45 min of this eruption, a massive atmospheric pressure wave and a series of tsunamis were generated and observed around the world (Carvajal et al., 2022; Imamura et al., 2022; Lynett et al., 2022; Omira et al., 2022). This event highlights an unexpectedly great hazard from volcanic tsunami worldwide (Paris, 2015; Whelan & Kelletat, 2003), which in Tonga’s case overprints an already extreme level of tectonic tsunami hazard (UNESCO/IOC 2020).

Due to the immediate post-disaster needs of the community and because of travel restrictions to and within Tonga caused by the ongoing COVID-19 pandemic, a comprehensive field survey of the impacts and effects of tsunami runup and inundation was not possible. Although local investigators of the Tonga Geological Services and Tonga Meteorological Service provided important information on the magnitude and effects of the tsunami immediately after the event, there was a lack of detailed and quantitative information on the impacts and effects of the tsunami in Tongatapu and outlying islands. To address this need, the Government of Tonga officially requested international technical assistance from Australia, New Zealand, and the UNESCO Intergovernmental Oceanographic Commission International Tsunami Information Centre (IOC ITIC), who obtained support from The Pacific Community (SPC). An international tsunami scientist (Dr. Jose C. Borrero) was engaged to provide remote training and assistance on post-tsunami field data collection techniques and to compile the results of a series of surveys into a technical report. Serendipitously, volcanologist Professor Shane J. Cronin of The University of Auckland was permitted entry to Tonga in March 2022 for an extended mission to conduct field investigations related to understanding ongoing volcanic threats. He undertook the tsunami field surveys and oversaw ground training with local scientists and staff from the Tongan Geological Services (TGS).

### Tonga Tsunami History

NOAA’s historical tsunami database (NGDC/WDC 2022) lists 32 tsunami events affecting Tonga (Supplementary Material Table S1). Of these, 27 have a validity ranking of ‘3’ or ‘4’ (likely or definite tsunami), four have a validity of ‘2’ (questionable tsunami) and one with a ranking of ‘1’ (doubtful tsunami). In terms of the tsunami source, 27 are associated with an earthquake, four with a volcanic source and one with a source listed as ‘unknown’. Of the four events attributed to volcanic activity, two are associated with the most recent activity on the Hunga volcano (January 13 and 15, 2022) with the others referring to events in August 1892 (Validity 3) and July 1907 (Validity 2), neither of which was significant in terms of tsunami effects.

The historical accounts describe significant tsunami and tectonic effects. For example, according to contemporary anecdotal accounts (Sawkins, 1856), on Christmas Eve 1853 a severe earthquake was felt on Tongatapu. The event reportedly led to widespread subsidence of the north-eastern portion of the island leading to ~ 3 km of inundation, while the western side of the island was uplifted by ~ 1 m. Interestingly, this record also describes an *“island having appeared about this time to the westward”*. This island was described by the captain of a whaling ship that had run aground on it “*as being only a few inches above the ocean (at a distance of thirty miles) and covered with black sand exactly like that on the shores of other volcanic islands in this and the Haabai [Ha’apai, sic] group”*. The wording of this anecdote suggests that the new island was located 30 miles offshore of Tongatapu, consistent with Hunga, lying ~ 35 nautical miles away. We also note that Soloviev and Go (1974) transcribe the words from Sawkins (1856) as “*the island was elevated about a decimetre (several inches) above the sea for a distance of 130 km (70 miles)”* which, besides the erroneous numbers, seems to suggest that the low lying island was 130 km long. We believe this translation implies a completely different meaning than that intended by the captain who is paraphrased in Sawkins (1856). Also noteworthy is that the Sawkins (1856) account describes inundation of the Hihifo Peninsula area in western Tongatapu in conjunction with the aforementioned earthquake. He writes *“I made particular inquiry of the natives of Tongataboo [Tongatapu, sic] if they had ever before seen any appearance of land in that direction, to which they replied. No,—but that it was their belief that it rose on the night of the earthquake (Christmas-eve, 1853), when the sea came over the land at Hihifo (the North Point)”*. This last point is important in that it is the locals assuming that the formation of the new island was associated with the 1853 earthquake–an event to which they would have no direct evidence. Thus, it is possible that the two events are entirely unrelated. Particularly given that Hunga volcano is known to periodically emerge and submerge as a result of volcanic activity and also generates many felt earthquakes on Tongatapu (e.g., 2014–15, 2021–22). It is also important that this earthquake occurred on Christmas Eve, a date that would likely be remembered by the local population who had largely converted to Christianity by the 1820’s. Even considering that this is also cyclone season, it is unlikely that the “encroachment of the sea for nearly two miles inland” on the north-eastern coast of Tongatapu was caused by a tropical storm as such an event would have been well remembered by the Tongans. Hence, the validity ranking of ‘1’ for this tsunami is probably too low in the light of what we now know from the 2022 event.

The earthquake of 1865, while strongly felt in the northern part of Tonga from Vava’u to Ha’apai only created a small local tsunami, although Okal et al. (2004) associated this event with tsunami accounts from Rarotonga and Nuku Hiva in the Marquesas Islands. As with the 1853 event discussed above, Okal et al (2004) describe how confusion over the historical record may have resulted in misinterpretation of the earthquake and tsunami effects. In this case it was the location of ‘Tau,’ an island where the ship *John Wesley* ran aground. At first, this was assumed by early accounts (Rudolph (1887) as cited in Okal et al., 2004) to be the easternmost island in the Samoa group; however, Okal et al. (2004) argue that it was more likely ‘Tau’, a small islet located 22 km northeast of Nuku’alofa. The more southerly location for a ship to have been grounded on a reef makes more sense when considering the earthquake location, as such an event would not have been strongly felt in Samoa. Furthermore, numerical modelling of the tsunami suggests that wave energy would have been projected to the SE and NW with much less projected northward towards Samoa.

An account from 1889 describes extreme waves from an unknown source that affected the eastern coast of Lifuka and Foa Islands in the Ha’apai group during the *‘gales of 7th and 8th March’*. Reports describe a coastal forest that had been swept clean of trees and shrubs to a height of 8 m *‘along the coast for miles’*, multi-ton boulders moved to elevations of 4–6 m above sea level, and areas of land being washed away with new beaches elsewhere. The reported heights were supposedly confirmed through ‘*scientific measurement by three gentlemen*’ who ‘*demonstrate[d] that [the waves] reached the extraordinary height of 36ft [11 m] above ordinary high-water mark*’. It is notable that the large waves occurred around the same time as a strong storm (likely a tropical cyclone given the time of year), however this is discounted as a possible wave source with the statement ‘*the waves could not have been caused by the gale simply, which was merely a heavy blow from the S.E., but must have been created by earthquake or other upheaval some distance from the group’*. The historical data also note that on March 16th the same storm wrecked several warships at anchor in Apia, Samoa and caused damage on shore in Savai’i ‘*to 15 ft. above high-water mark only’*, significantly less than the 36 ft measured in Tonga. Nevertheless, this event remains unexplained as there is no confirmed earthquake or volcanic event either in the near- or far-field that could be responsible for the wave.

Maximum tsunami amplitudes from the historical events in Tonga have rarely exceeded 2 m (Okal et al., 2004, 2011), apart from ~ 10 m tsunami heights from the mysterious 1889 event, ~ 20 m on Niuatoputapu from the well-documented September 2009 event (Fritz et al., 2011; Okal et al., 2010) and the extreme effects from the most recent Hunga source. In the modern instrumental era, the Nuku’alofa tide gauge has recorded a tsunami amplitude greater than 0.5 m only once, in 2011 following the Great Tohoku earthquake. It is interesting that neither the 1868 Arica nor the 1960 Valdivia tsunamis, which were widely recorded throughout the Pacific, are mentioned, but effects from much smaller events, such as the 1877 Iquique earthquake are. Numerical modelling suggests that tsunami amplitudes of ± 1.5 m and ± 1.0 m would be expected at Nuku’alofa for the 1868 and 1960 tsunamis (Borrero, unpublished). Beyond the historical accounts, investigations into prehistoric tsunami events in Tonga include the discussion around the presence of numerous large and anomalous boulders located on Tonga’s western coast, presumed by some to have been deposited by an extreme tsunami (Frolich et al. 2009). Additionally, Lavigne et al. (2021) provide physical and cultural evidence suggesting the occurrence of a large tsunami event affecting Tonga in the fifteenth century. However, no causative mechanism for either of these events has been unequivocally identified.

### Field Survey Details

Tsunami runup and inundation surveys were conducted on Tongatapu, the Ha’apai and ‘Eua between April 4 and 17 June 2022 and on Tofua in October 2022. The tsunami runup and inundation survey was conducted as per standard methodologies (i.e. IOC/UNESCO 2014). Data was collected by visiting the tsunami affected sites and measuring coincident transects of local topographic elevation and tsunami flow traces along each transect. Survey lines were run perpendicular to the coast and in each site a minimum of two and up to four survey lines were measured. Position and elevation data were collected using survey grade RTK GPS receivers and heights were corrected to the Tongatapu local mean sea level on Tongatapu and Eua. On the Ha’apai islands tsunami heights were measured directly by difference along GPS survey lines and checked against satellite LiDAR based measurements where available (the latter courtesy of James Garvin, NASA). While the positional precision for the measurements was generally within a few centimetres, the overall accuracy of the absolute heights may vary up to several tens of cm, partly due to measurement error, but also to local irregular topographic conditions along the coastlines.

Surveys were collected from the most-recent high-tide mark (wetted limit or detritus limit) up to the interpreted run-out of the tsunami. Maximum runup was identified by floated debris, evidence of wetting through staining or vegetation die-back, deposits (rarely), and most commonly, by floated pumice, which formed distinctive marker lines. Many of the coastal soils in this area have abundant pumice with pumice from recent eruptions (e.g., Le Havre) lying on reefs, lagoons and within beach deposits. In addition, pumice rafts from the December 2021 and early January 2022 eruptions of Hunga would have arrived at shore by ~ 12 January. Hence pumice tide-lines from the tsunami included pumice mixed from several pre-15 January sources. Survey transects were targeted, where possible, to areas where true runups could be measured (i.e., un-affected higher slopes beyond the tsunami runup). Transects were also targeted to maximise the possibility for flow-height measurements–e.g., by choosing places where trees were standing. In areas of near-total destruction, typically only a few coconut trees remained. Flow height measurements were taken from trees or other tall obstacles either directly along the transect line or projected from up to 20 m perpendicularly on either side of the line. Flow markers included, in order of reliability: rafted/floated debris within trees, impact/damage marks on bark/trees/structures, freshly broken tree branches, debris piles, stain marks on walls and debris lines. Where possible residents from the survey areas were interviewed and were able to point out many additional flow-height markers that they had seen soon after the event. Flow heights were estimated using a laser-range finder. Flow height measurements have inherently great variability due to the serendipity of the presence of trees, whether they were distinctively marked and whether the marks represent the maximum flow height. Hence, in most cases the flow-heights are considered a minimum value of the true water level.

Due to the relatively small tide range (~ 0.75 m typically), the data presented here have not been adjusted to account for the tide level at the time of the tsunami. However, generally, the tide level at the time of the tsunami was approaching high tide, thus the tsunami heights reported here could be reduced by ~ 0.4 m. However, given the relatively large tsunami heights and the uncertainty inherent in interpreting the flow traces this correction can be neglected at some sites. At other sites, such as northern Tongatapu where the tsunami heights are smaller, the 0.4 m correction is more important.

### Tide Gauge Records

**Fig.2:**
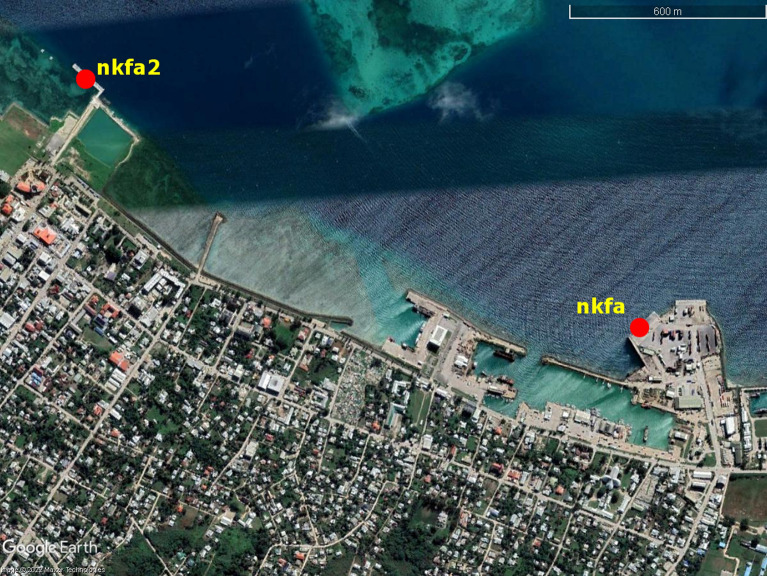
Locations of the nkfa and nkfa2 tide gauges along the Nuku’alofa waterfront

**Fig. 3:**
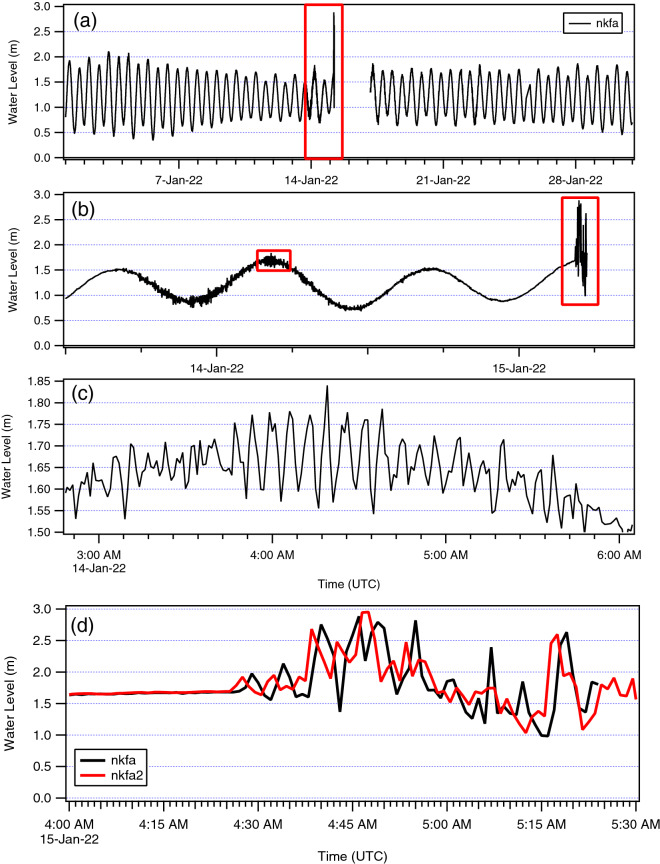
Tide gauge water level data from Nuku’alofa. Panel **a** shows data from the month of January 2022, note the gap in the record commencing shortly after the tsunami arrival. Panel **b** shows an enlargement of the time span indicated with the red box in A. Small tsunami activity can be seen commencing at ~ 1800 h on from 13 January 2022. Red boxes indicate areas enlarged in Panels **c** and **d**. Panel **c** shows a close-up of this early tsunami activity with tsunami amplitudes of ± 15 cm. Panel **d** is a close-up of the main tsunami on 15 January with the data from nkfa2 included

Two tide gauges were in operation on the Nuku’alofa waterfront during the tsunami, the ‘nkfa’ sensor at Queen Salote Wharf and ‘nkfa2’ located 1.8 km to the west at the Vuna Wharf (Fig. 2). Water level data was also recorded at Neiafu on Vava’u some 300 km north of Nuku’alofa. In Nuku’alofa, at the time of the tsunami, only the ‘nkfa’ (Queen Salote Wharf) sensor was transmitting data to the Tonga Meteorological Service, which serves as Tonga’s National Tsunami Warning Center, and the IOC Sea Level Monitoring Facility website in real time (https://www.ioc-sealevelmonitoring.org/) and this gauge failed during the tsunami at 0525 UTC as shown in Fig. 3. After the event, the Australian Bureau of Meteorology (BoM) released the complete data from the station at Vuna Wharf (nkfa2). Inspection of the data from the day before the main eruption shows a period of sea level agitation commencing at approximately 1800 h on 13 January (UTC) and lasting for approximately 24 h. The main tsunami event commenced at approximately 0425 h (UTC) on January 15 with the highest tsunami water level reaching just under 3.0 m relative to the tide gauge datum. The data from nkfa and nkfa2 closely mirror each other with the tsunami signal on nkfa2 slightly preceding that of nkfa due to its more westerly location.

**Fig. 4:**
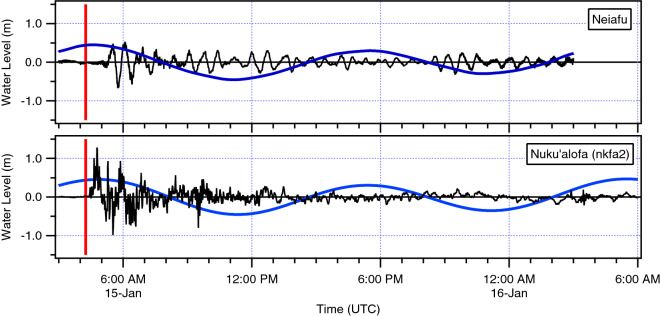
The detided (black) water level and the predicted tide (blue) at Neiafu (top) and Nuku’alofa (bottom). Vertical red line indicates the time of the large explosion from the erupting volcano (0415 UTC)

**Fig. 5:**
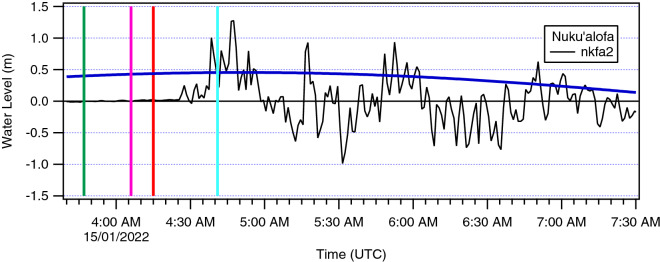
Detail of the first 3.5 h of tsunami activity at Nuku’alofa. Vertical lines indicate key times; green (0347) the start of the surface eruption, pink (0406) eruption plume visible on satellite imagery, red (0415) the time of a large explosion and cyan (0441) the ~ 26-min tsunami arrival time for the large eruption from Hunga to Vuna Wharf

The Neiafu and Nuku’alofa (nkfa2) data sets were detided using a predicted tidal curve derived from tidal constituents at their respective stations (Fig. 4). The figures show that the main tsunami of January 15th occurred during the high tide period on Tongatapu and shortly after high tide at Neiafu. At Neiafu tsunami amplitudes were generally smaller than the tide range, while at Nuku’alofa the tsunami amplitudes are more than twice the tide range. The plots also show the rapid onset of tsunami activity at Tongatapu and the ~ 1.5 h travel time to Neiafu. Also, at Neiafu we see that the tsunami signal is more periodic in nature, suggestive of resonance in the complex bathymetry and dendritic channel characteristic of southern Vava’u, where the tide gauge is located. Figure 5 shows the first ~ 3.5 h of the de-tided tsunami record at Nuku’alofa (nkfa2). In this plot the vertical red line is the time of the large magnitude explosion of the eruption sequence (USGS, 2022), which occurred at 0415 UTC. Tsunami activity is present on the gauge at ~ 0425 UTC, just 10 min after the explosion–which is too early for water waves generated by it. The implications of this timing are considered further in the Discussion section below.

### Field Data Summary

**Fig. 6:**
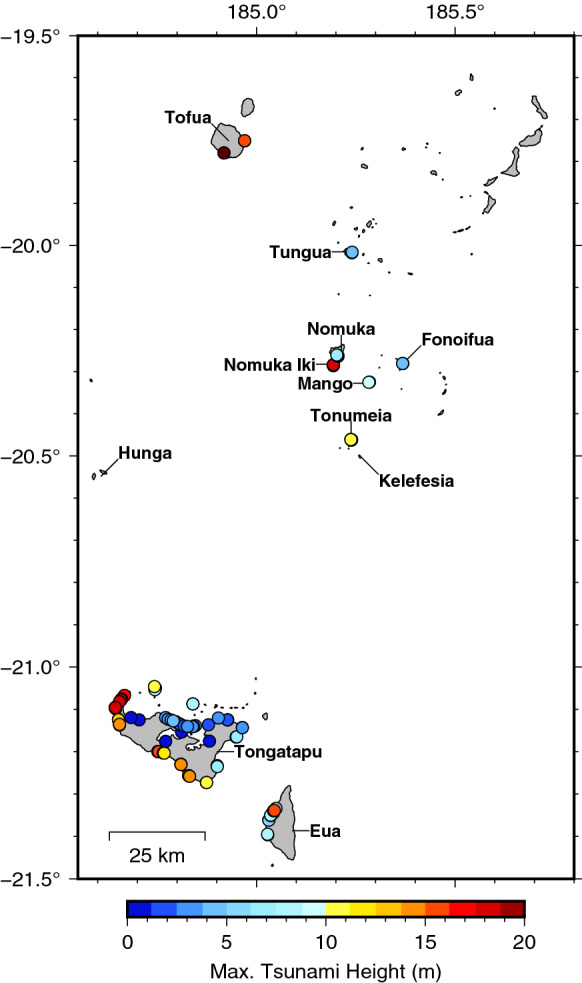
Summary data plots of maximum measured tsunami heights

The measured tsunami data points are presented in Supplementary Material Table S2(a,b,c). This data, along with detailed transect data from each site, is also available in an associated technical report (Borrero et al., 2022). The overall maximum measured tsunami heights are plotted against location in Fig. 6 below. On Tongatapu, tsunami waves caused catastrophic damage to the western part of the island with runup heights greater than 15 m along the Hihifo Peninsula from Ha’atafu south to Utukehe with maximum measured total tsunami heights of 18–19 m in the vicinity of Kanokupolu and Liku’alofa. Inundation distances varied greatly ranging from less than 200 m on steeper coasts where there was no overtopping, to more than 1000 m where the tsunami overtopped and inundated across the entire peninsula. In Nuku’alofa media reports showed videos of waves crashing over sea walls and flooding houses, suggesting tsunami runup heights of the order of 3–5 m, heights that were confirmed during this survey. The southwest facing coast of Tongatapu experienced tsunami runup heights of 10 to 15 m with inundation distances generally less than 100 m due to the steep, cliff and terrace topography. Despite facing directly away from the tsunami source, sites on the east coast of Tongatapu nevertheless experienced tsunami heights greater than 6 m with inundation distances varying from ~ 20 m at Emeline to ~ 80 m at Halaika. On Eua the survey was constrained to the central west coast. Tsunami runup heights were generally of the order of 4–8 m with one measurement up to 18 m. In the Ha’apai island group, the survey team visited the islands of Tungua, Nomuka, Fonoifua, Mango, Nomuka Iki and Tonumea, focussing on the inhabited areas of the islands. Tsunami effects were severe with the largest tsunami height of > 20 m measured on Nomuka Iki where surges washed over low-lying areas causing extreme erosion and the complete stripping away of coastal forests; effects reminiscent of those seen in the Mentawai Islands after the October 2010 earthquake and tsunami (Hill et al., 2012) and on islands in the Sunda Strait following the tsunami generated by the eruption and flank collapse of Anak-Krakatau volcano in December 2018 (Borrero et al., 2020). The team also visited Tofua where tsunami runup heights of ~ 20 m were measured on the southern coast.

### Western Tongatapu and the Hihifo Peninsula

**Fig. 7:**
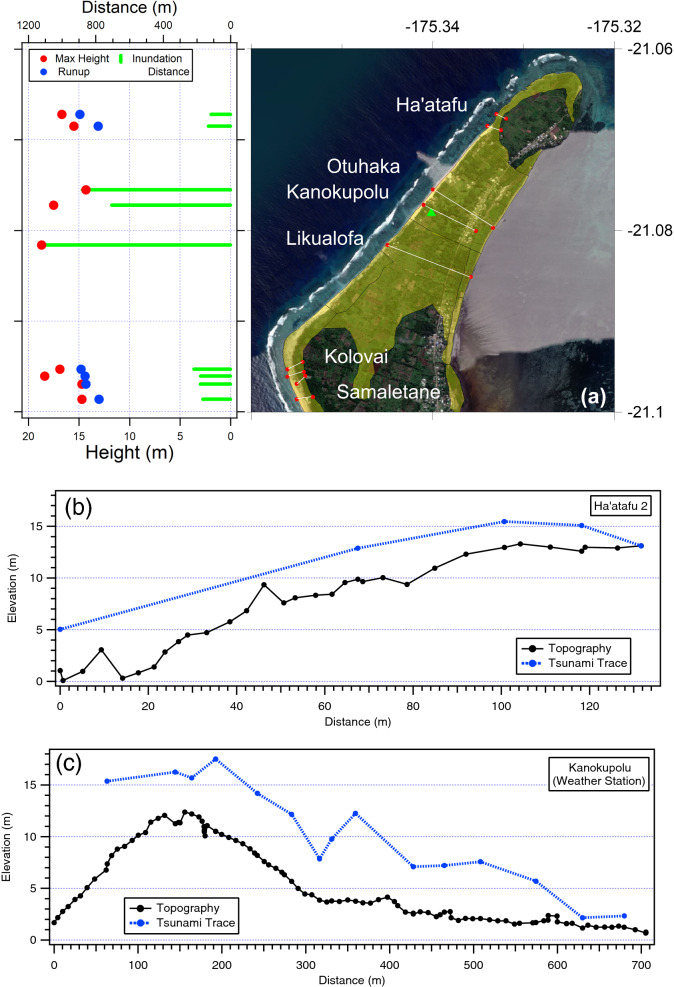
**a** Locations surveyed along the western coast of Tongatapu. Yellow shaded area indicated extents of inundation. Green tringle indicates the location of the Tongan Meteorological Service weather station. The start and endpoints of each transect are indicated with the red dots. Left plot shows maximum tsunami trace height, maximum runup height and maximum inundation distance along each transect. **b**, **c** Detailed transects from Ha’atafu and Kanokupolu

Some of the most extreme tsunami effects occurred in the Kolovai District of western Tongatapu where tsunami surges overtopped the narrow Hihifo Peninsula causing near total destruction along the shoreline and inundating over distances of up to 1 km (Fig. 7). At Kanokupolu, a weather station co-located on a cellular phone tower situated at approx. + 13 m elevation was toppled by the tsunami, ripped from its foundations and transported more than 200 m from its original location. The weather station collected data at 10 min intervals and this data was uploaded every hour on the hour via satellite telemetry. The station made its final transmission at 0500 UTC (18:00 local) and was therefore destroyed some time after (Fig. S1).

**Fig. 8:**
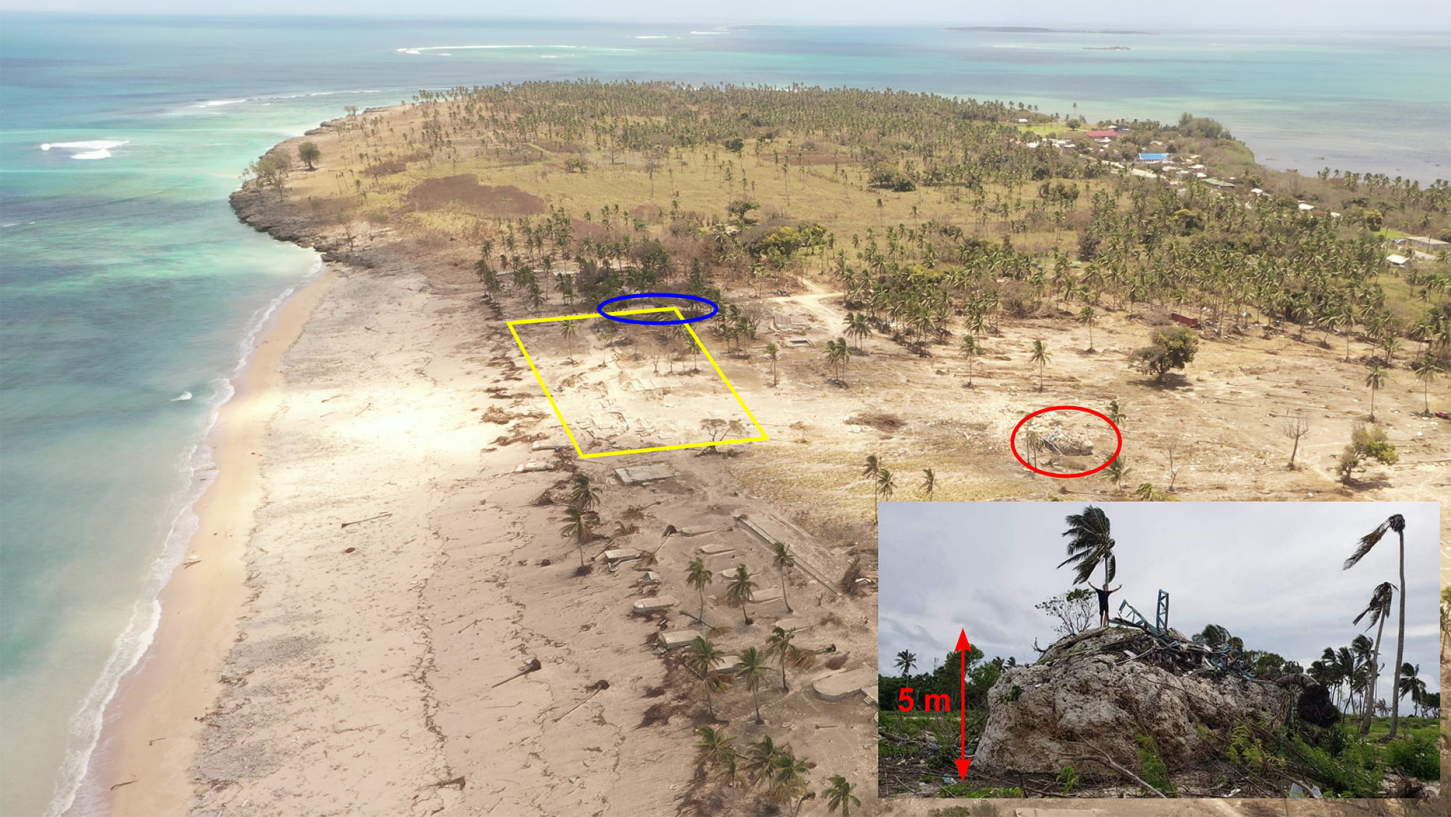
View to the north along the the Ha’atafu section of the Hihifo Peninsula. Yellow rectangle indicates the site of Ha’atafu Beach Resort. Blue ellipse is the location of the car videos mentioned in the Ha’atafu Timeline section. The red ellipse is the location of the ‘tsunami rock’, shown in the inset after the tsunami and covered with debris indicating that it had been overtopped by the waves

Ha’atafu was the scene of remarkable tales of survival (Fig. 8). Moana Paea, proprietor of the Ha’atafu Beach Resort described hearing a commotion from the beach area as the first surges rose above the high tide level, reaching the beachside boundary of the resort. At the same time, Australian High Commissioner Rachael Moore, who was at the resort with her family, observed and photographed these early surges indicating a time of ~ 0415 UTC (17:15 local) for the first significant waves. Videos shared on social media also show local residents who had been spending the day on the beach, returning to their cars after being chased off the beach by the first surges. Ms. Paea and her staff began alerting other guests to leave the resort, all of whom went to the carpark and began to leave by vehicle. A second video obtained from social media shows the vehicles evacuating as a tsunami surge rushes up the road adjacent to the resort, toppling a fence as it progresses inland and narrowly missing a car full of people that had been parked on the waterfront. As this was going on Ms. Paea, her family and staff, noting the vehicular congestion, fled on foot in search of a large ‘tsunami rock’ they had previously identified as high ground for potential tsunami evacuation. While looking for this rock in the heavily vegetated bush area behind the resort, they felt and heard one of the large explosions and shock waves released during the eruption, noting that ‘it felt like a bomb’ and nearly ‘knocked [us to the] ground’. Unable to locate the ‘tsunami rock’ the group continued by foot to the lagoon side of the peninsula seeking refuge with others on the roof of a house. Their inability to locate the tsunami rock was fortuitous in that the tsunami surges completely overtopped the rock leaving it covered with debris (Fig. 8).

### Nuku’alofa

**Fig. 9:**
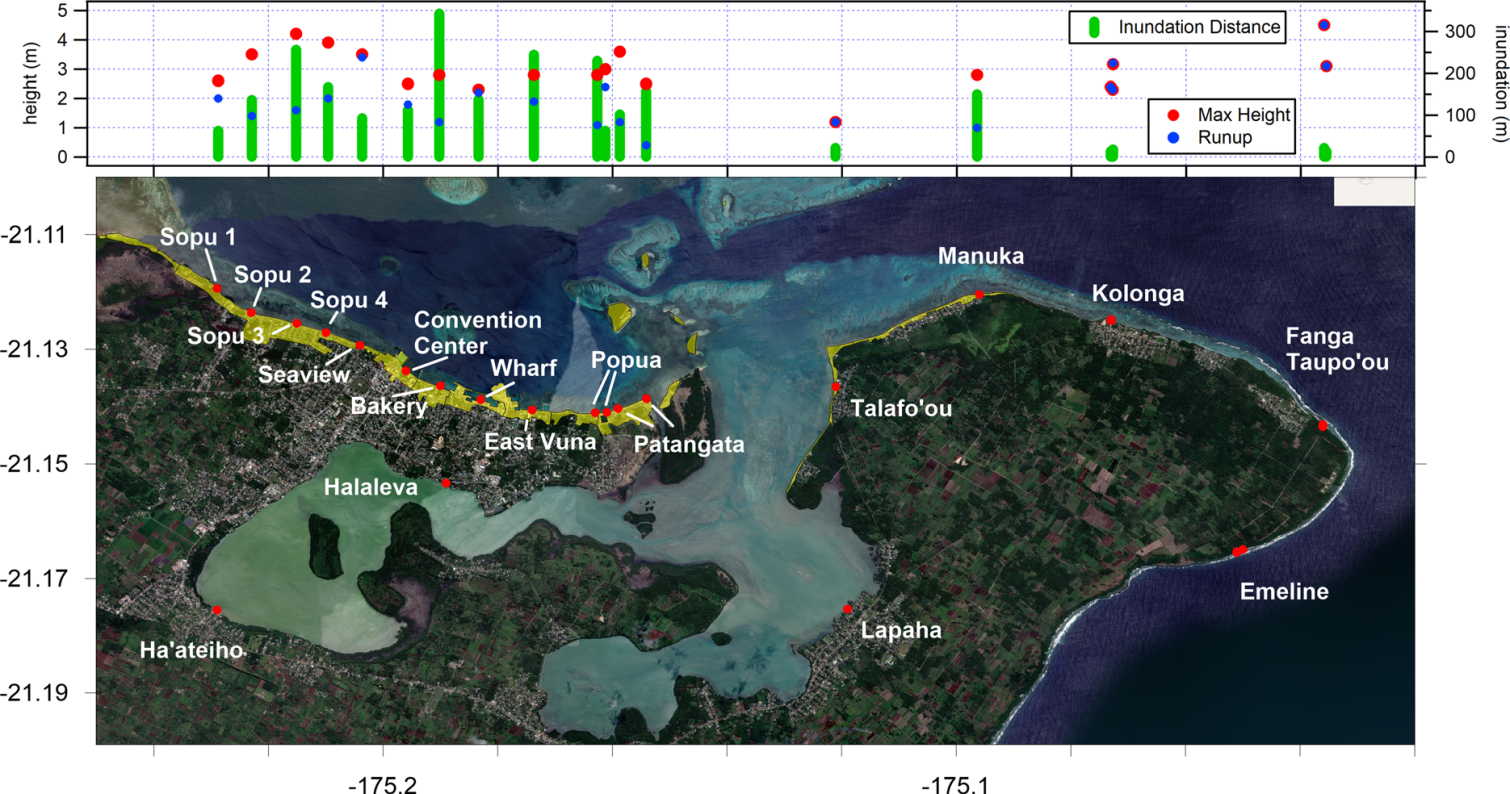
Maximum tsunami trace elevation (red) and run up height (blue) along the north coast of Tongatapu

**Fig. 10:**
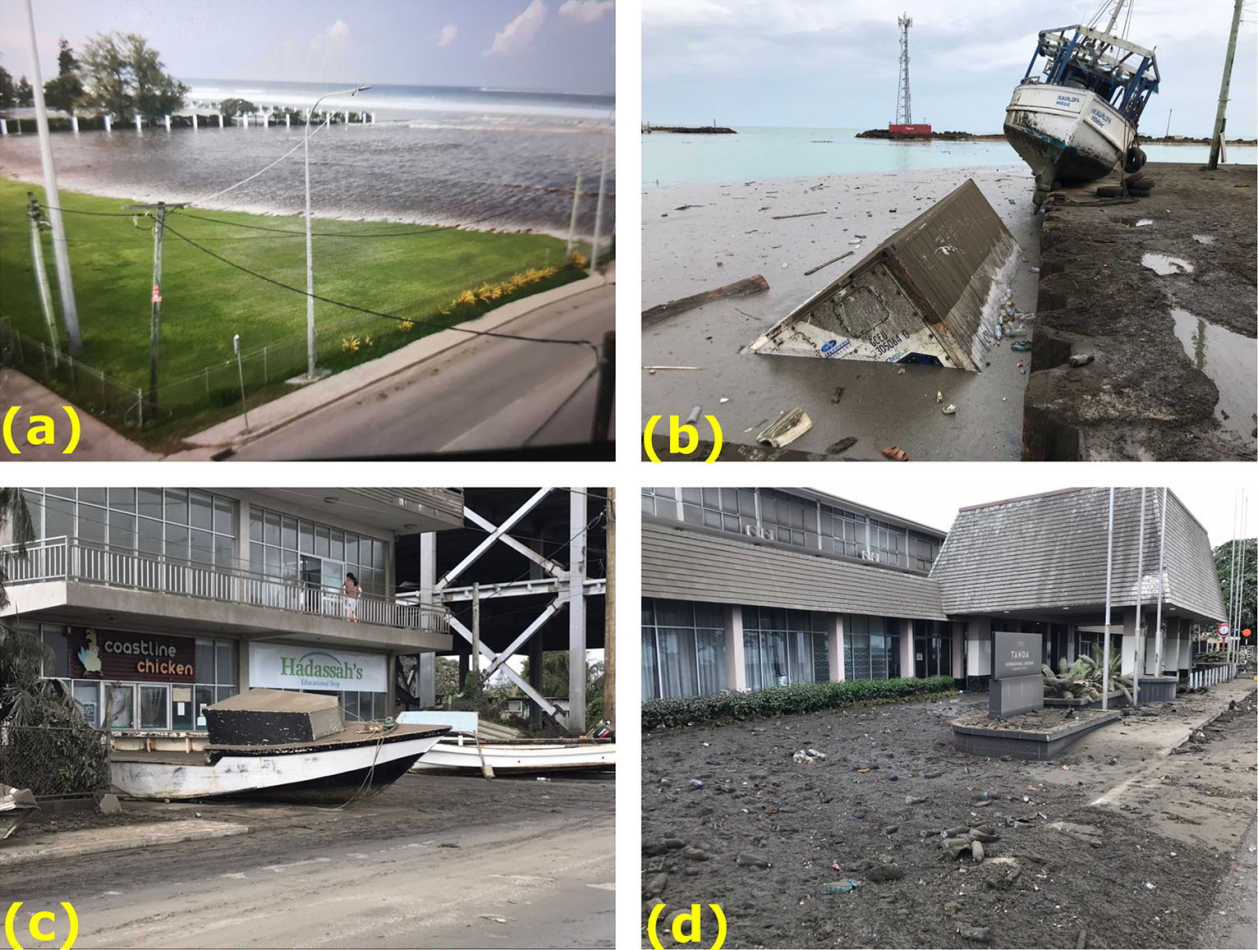
Scenes from Nuku’alofa **a** tsunami surge coming ashore on the grounds of the Royal Palace at 17:47 local time **b**, **c** smaller vessels floated on to the wharf or across the street **d** inundation at the Tanoa Hotel along the waterfront

Tsunami heights along northern coast of Tongatapu in the vicinity of Nuku’alofa were generally in the range of 2–4 m with inundation distances of 100 to > 300 m (Fig. 9). East of the entrance to Fanga’uta Lagoon inundation distances dropped of significantly however measured maximum tsunami trace hights remained consistent with sites to the west. While there was no major structural damage to the port facilities, several small boats were floated out of the basin and were deposited on dry land and several shipping containers and boats washed back and forth within the port basin. The waterfront roadway in the port area was covered with debris and the entire area was affected by a thick layer of volcanic ash (Fig. 10).

### Ha’apai Islands

**Fig. 11:**
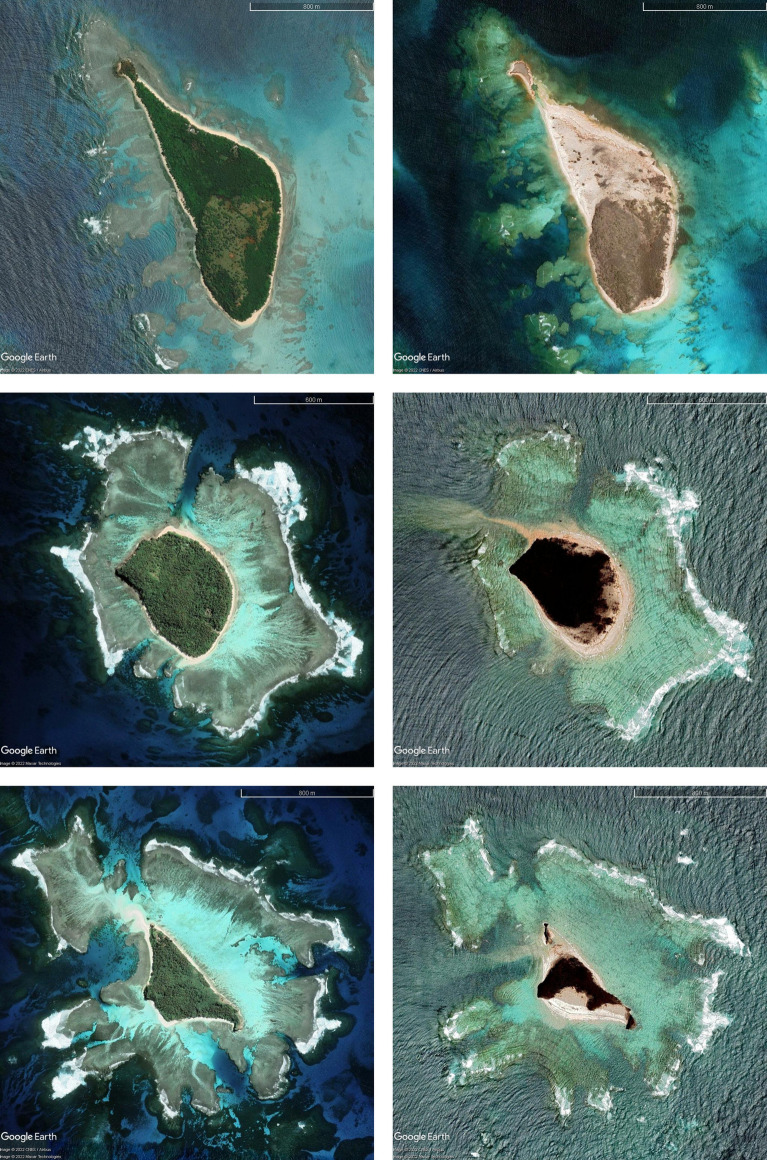
Before and after images of Nomuka iki (top) Tonumea (middle) and Kelefesia (bottom) showing large scale morphological change due to tsunami over wash. (Imagery from Google Earth, Maxar Technologies)

**Fig. 12:**
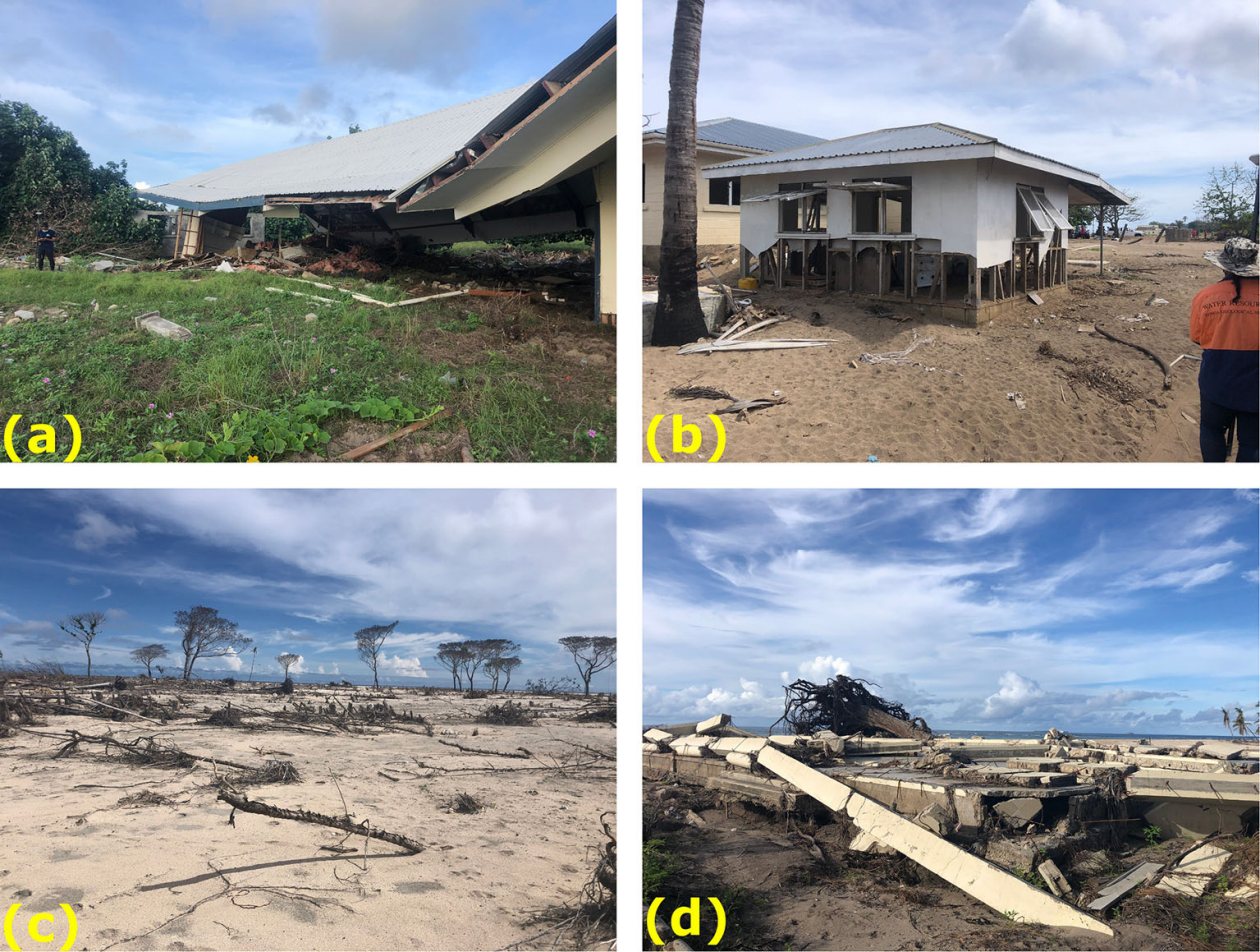
Tsunami damage on Tungua **a** Nomuka **b**, Nomuka Iki **c** and Mango **d**

The survey team visited several islands north of Tongatapu (see Fig. 6). While all the islands visited were affected by the tsunami, the most severe effects and largest tsunami heights were observed on Nomuka-Iki, Mango and Tonumea Islands. Each of these islands, as well as Kelefesia (which was observed only from a boat 1 km offshore), were completely over washed by the tsunami surge. The extreme inundation resulted in drastic changes to the coastal morphology and shape of these islands (Fig. 11), effects that at first glance seem more extreme and erosive in nature than those described by Kench et al. (2006) on Maldivian atolls after the 2004 Indian Ocean tsunami. At just under 5 m, the maximum measured tsunami heights on Tungua were smaller relative to the other islands, however these measurements were taken at the village on the on the eastern side of the island which faces away from the direction of tsunami approach and was protected by a large shallow reef. Nevertheless, there was severe damage to modern buildings and robust structures, such as a solar power installation (Fig. 12a). On Nomuka, tsunami heights in the village were in excess of 8 m with ~ 6 m runup and > 100 m of inundation (Fig. 12b). Nomuka-iki, a small island located 2 km to the southwest, partly protected Nomuka, and was hit much harder, with complete over wash of the lower lying part of the island and measured runup of up to 20 m along the western face of a topographic high on the southern half of the island. The low-lying forest on the northern half of the island was stripped virtually clear to the subsoil (Fig. 12c), reminiscent of the destruction seen on Painatan Islands in Western Java following the 2018 tsunami generated from the eruption and subsequent flank collapse of Anak Krakatau (Borrero et al., 2020). There were tsunami heights of the order of 5 m on Fonoifua with damage to several buildings and the solar power facility. Finally, the village on the northern coast of Mango Island was totally destroyed by tsunami runup exceeding 7 m and maximum measured tsunami trace heights of nearly 10 m (Fig. 12d). In early-October 2022, a survey team visited Tofua and measured tsunami runup of > 20 m on the southwest coast and 14–16 m on the east side of the island. At these sites the inundation distances were relatively short (35-55 m) due to the steep topography.

People interviewed by the field survey team (including the village “town officer” or their representative) during the visits to the inhabited Ha’apai islands (Nomuka, Mango, Fonoifua and Tungua) all describe the rapid retreat of all people to high ground on foot upon the first tsunami waves arriving. In all cases these were unsheltered areas of gardening land located a few tens of metres above sea level and 100 to 500 m inland. Onset of darkness and heavy ashfall, along with no direct line of sight through trees to the coast prevented detailed timelines to be established of the wave train. When the sounds of waves and destruction were silent for at least an hour on both Mango and Tungua, the respective town officers organised scouting parties of men to descend and check for damage (and in the Mango case search for a missing person). In both cases ongoing smaller tsunami surges were reported. On Tungua this party arrived near the coast after ~ 0900 UTC to witness the arrival of a later tsunami wave that inundated low-lying areas approximately one quarter of the maximum runup.

## Discussion

The field data presented here provides valuable insight into the tsunami hydrodynamics that occurred along near-source coastlines. These observations provide clues relating to the timing and tsunami source mechanisms. This is important because many different processes likely contributed to wave generation (Lane, 2022; Lynett et al., 2022). The field survey also provides information on factors which contributed to the extraordinarily low number of casualties.

### Observations from the ‘Ngahau Siliva’

**Fig. 13:**
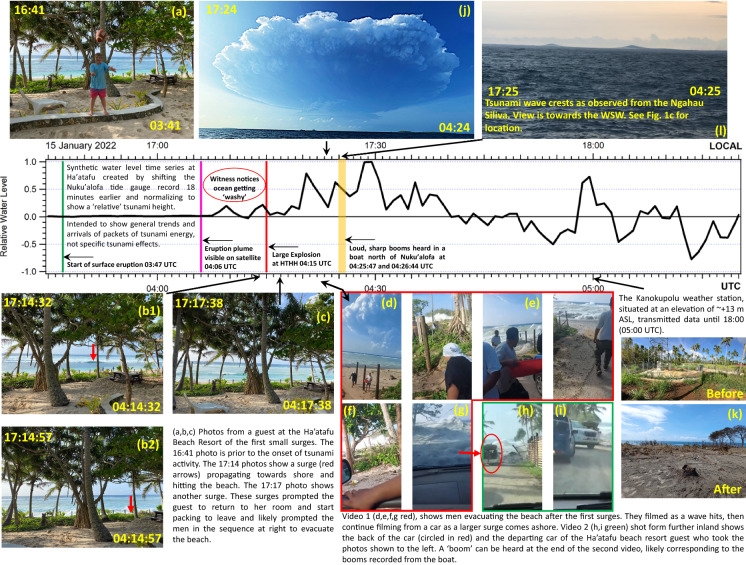
Timeline of events at Ha’atafu and other locations. Times given as UTC and Local (UTC + 13). Descriptions given in the figure and in the preceding text

At the time of the eruption on January 15, the Tongan Navy vessel Ngahau Siliva was *en route* from Mango to Nuku’alofa after delivering water and supplies to the island which had been affected by ash fall from the eruption of the previous day. At 0411 UTC, approximately 2 h after departing Mango, the crew heard one large explosion from the volcano. At 0425 UTC (17:25 local) they saw two large wave crests, estimated at 9–10 m amplitude off the starboard (right) side of the vessel in a direction roughly south of west (Fig. 13l and Supplementary Material Video 1). The position of the vessel at the time of this observation (Fig. 1c) shows an area of shallow bathymetry (< 100 m depth) in the direction of the waves. By 0450 UTC they reported complete darkness with heavy ashfall and pumice falling from the sky. At 0525 UTC, located 36 km southeast of their previous position (Fig. 1), the boat’s engines failed due to the ash, and they drifted for several hours while repairs were made. During this time crewmembers reported feeling as if they were riding on the waves but were unable to count a specific number of crests. After restarting the engines at 1000 UTC (11 pm), the vessel changed course and headed north to Vava’u to await further orders.

### Pre-15 January 2022 Tsunami Waves

One of the largest eruptions of the 20 December 2021–15 January 2022 volcanic sequence was photographed and observed by TGS staff on the day before the climactic eruption. Tsunami activity was recorded on the at Nuku’alofa tide gauge commencing at approximately 1600 UTC on 13 January (05:00 local on 14 January) and continued for some 18 h before settling down in the evening, leaving the calm conditions observed on the morning of 15 January. While Lane (2022) speculates that a flank collapse of the central cone joining the island of Hunga Tonga and Hunga Ha’apai could have contributed to this activity, that mechanism would not account for the long duration and periodic nature of the signal. Instead, over this time frame, we see nearly continuous tsunami activity with amplitudes in the range of ± 10–20 cm and relatively short periods of ~ 5–7 min (see Fig. 3). It was during this activity that the Tonga Meteorological Services issued a Marine Tsunami Warning, which was consistent with observations of strong currents at the entrance to Nuku’alofa Harbour (see Supplementary Material, Video 2a, b). This type of activity could alternately be generated by submarine volcanic processes, particularly faulting and caldera motion caused by decompression of the magmatic system following the largest eruption of the sequence to this point.

### “Early” 15 January 2022 tsunami

During the main tsunami event of 15 January, ‘early’ arriving waves occur on the tide gauge record. Tsunami arrival times along the coastline of Tonga can be determined analytically based on the shallow water approximation, or through numerical modelling, with both approaches strongly dependent on the accuracy of the bathymetric model. Using a nonlinear, dispersive hydrodynamic code, Lynett et al. (2022) predict tsunami travel times of ~ 8 min to Ha’atafu and the western Hihifo Peninsula and ~ 26–28 min to central Nuku’alofa waterfront (King’s Palace/Vuna Wharf to Queen Salote Wharf). The 26-min arrival time relative to an assumed 0415 UTC source origin time (USGS, 2022) is plotted in Fig. 5 showing significant tsunami activity prior to the predicted arrival time suggesting that tsunami waves were generated by other mechanisms prior to the large explosion at 0415 UTC. Based on the arrival time at Nuku’alofa, these early waves would have been generated at approximately 0400 UTC and arrived on the western facing beaches of northwest Tongatapu at 0408; seven minutes before the 0415 UTC explosion occurred; timing that is consistent with the eyewitness accounts discussed below.

### Ha’atafu Timeline

We use eyewitness accounts, photographs, instrumental data and other information to recreate a timeline of events occurring at Ha’atafu and other locations on Tongatapu (Fig. 13). The timeseries shown is the Nuku’alofa tide gauge record shifted 18 by minutes to match approximate arrival times at Ha’atafu. The time series has been normalised by the maximum amplitude on the record to show relative tsunami heights with the intent to simply to use this record as a guide to correlate with the information obtained from eyewitnesses. The first photograph (Fig. 13a, taken at 0341 UTC) was taken from the Ha’atafu Beach Resort before the resort guests noticed any unusual wave activity. A small bore advancing towards the beach alerted the witness to danger who then took two photos of the surge coming ashore at ~ 0414 UTC and reflecting off the steep beach face (Fig. 13b). A third photo taken at 0417 UTC shows the approach of another small bore (Fig. 13c).

At around this same time, a group of young men spending a day on the beach were a few hundred meters north of the resort. Video recovered from social media (Supplementary Material Video 3) shows the group leaving the beach and commenting about being affected by a surge (Fig. 13d). As they reached their cars, a larger surge came ashore forcing them to scramble to high ground (Fig. 13e). This surge did not reach the level of the roadway where the cars were parked. After one of the cars leaves, the video resumes, now being shot from inside a second car (Fig. 13f). The scene unfolds with a view to the north as a large surge of white water is seen advancing towards them. The driver reverses as the now larger surge comes ashore, splashing over the front of the car (Fig. 13g). As they retreat, a second video (Supplementary Material Video 4a, b), shot from a third car further inland shows the back of the second car (with hatchback open) retreating as the wave topples a fence (Fig. 13h). In this sequence, two other cars are seen entering the frame from the left and turning right, away from the ocean, one of which contains High Commissioner Rachael Moore, who took the photos of the first surges (Fig. 13i). At the very end of the second video, an explosion can be heard, which we interpret to be the same explosions that were recorded on camera at 0425 to 0426 UTC by people fishing from a boat approximately 7.5 km ENE of Nuku’alofa (Fig. 13j, Supplementary Material Video 5). While other explosions can be heard somewhat earlier at ~ 0422 UTC from another video recorded in Nuku’alofa (Delmar, 2022), we do not believe this would have allowed enough time for Ms. Moore, who took her last beach photograph at 0417, to have returned to her room, changed clothes and begin packing, before being alerted by the Ha’atafu Beach Resort staff and her husband to immediately evacuate. They then moved to the cars, loaded the family and pulled out of the resort with their departure captured on the second video clip. A time of 8–9 min (0417 to 0426 UTC) is more plausible than just the four minutes to 0422 UTC.

At the time the cars are seen evacuating, Ms. Paea and her family and staff from the resort were evacuating on foot trying to find the ‘tsunami rock’. It was at this time that they heard and felt the large explosion. Unable to find the rock, they carried on by foot and located a stand of mango trees that some of them climbed up. After receiving a phone call from a family member who was *en route* by car to pick them up, they came down from the tree and ran to the roadway on the bay side of the peninsula. There they took refuge with other locals on the roof of a house before being picked up and driven away. It was not until *just after* they were picked up and out of the area, that the largest tsunami surge came across the peninsula inundating the Ha’atafu and Kanokupolu villages.

This sequence of events is supported by the hypothetical water level timeseries presented in Fig. 13 which shows surges of increasing size arriving between 0409 and 0426 UTC. What is less clear is whether or not the large peak seen at 0429 UTC overtopped the peninsula. Based on the account from Ms. Paea, it would seem that it didn’t, since her group was able to get to the highway and took shelter for a number of minutes before being picked up in a car and it wasn’t until after they left that a surge crossed the peninsula. This is also supported by the fact that the weather station at Kanokupolu (Fig. 13k) transmitted data at 0500 UTC and its rain gauge, located at 0.6 m above ground, did not record the presence of any water (Fig. S1). As noted previously, the weather station collected data at 10 min intervals and uploaded every hour on the hour via satellite telemetry. The data transmission is very sensitive to the antennae orientation and would have been unlikely to operate if disturbed (Chandra A., *pers. comm.*). Hence, this strongly suggests that the weather station was not toppled by a tsunami surge until sometime after 0500 UTC. However, we reiterate that the timeseries shown in Fig. 13 is based on data recorded at Nuku’alofa and the relative heights and timing of individual wave peaks may be quite different than what actually occurred on the west coast, however, larger scale features of the record may be consistent between the two locations.

After the main surges, later, smaller tsunami are implied from both eye-witness accounts on Ha’apai (as described above) and observations of the field team. The Tungua Town Officer observation was of a wave inundating after ~ 0900 UTC. At ~ 0900 UTC Mr. Branko Sugar arrived back to Nukualofa harbour after riding out the tsunami in deep water between Tongatapu and ‘Eua in his 9 m game-fishing vessel. He reported the resumption of loud rumbling explosions from Hunga at this point, so he and his crew quickly drove home through the damaged streets. During the field survey, at several sites on the western Hihifo Peninsula, a “late” tsunami runup is evidenced by ashfall stratigraphy. The main tsunami deposits are coated in ~ 2–3 cm of ashfall, whereas the inundation and deposits of the latest event(s) had little to no ash cover suggesting runups of only ~ 2–3 m.

Efforts to model the tsunami from source (Bosserelle et al., 2022; Grilli et al., 2022; Lynett et al., 2022; Pakoksung et al., 2022) generally produce one large surge along the west coast that overtops the peninsula (or not, depending on the quality of the topography used in the model), presumably destroying the weather station. However, this occurs too early for the station to have been able to transmit its final data at 0500 UTC (6 pm local) and does not reconcile with the experiences of the eyewitnesses. These same models, however, produce reasonable fits to water level records from Nuku’alofa and other gauges in the region (i.e. DART tsunameters or coastal gauges in New Zealand and elsewhere). The uncertainty in the source model and veracity of attempts to model the detailed hydrodynamics is further underscored by recent work of Heidarzadeh et al. (2022) who modelled the tsunami source as a positive Gaussian bulge on the water surface, or in other words, the direct opposite of the approach used by the previously mentioned studies where the tsunami source is modelled as a depression the ocean surface. Yet the modelling of Heidarzadeh et al. (2022) also somehow manages to produce an acceptable fit to measured data at the New Zealand DART stations, although they did not compare their output to coastal tide gauge records from Nuku’alofa or elsewhere. Thus, the timing of the overtopping surge or surges in western Tongatapu and the timing of the destruction of the weather station, not to mention the details of the tsunami source itself, remain an enigma.

### Low Number of Casualties

Despite the total destruction of several beach resorts along western Tongatapu and significant inundation along the populated northern coast, only four deaths were attributed to the tsunami. This low number of casualties can be attributed to multiple factors including: the event occurred during the day, the early arrival of moderate tsunami waves prior to the largest and most destructive waves, the marine tsunami warning issued the day prior by Tonga Meteorological Services, the lack of tourists in Tonga, and the effectiveness of tsunami awareness education and outreach campaigns conducted since the 2009 Samoa-Tonga tsunami hit Niuatoputapu, Tonga causing nine deaths.

The occurrence of the tsunami during daylight hours on a sunny weekend afternoon likely helped to reduce the number of casualties. People were out and about, generally aware of their environment and able to react despite the lack of at least one of the normally discussed ‘natural warnings’ associated with tsunami disasters, i.e. strong ground shaking. In addition, there was a generally heightened awareness of the possibility of tsunami, because in the previous few weeks several eruptions of Hunga were witnessed and reported in news media. This is in contrast to the effects of the 2010 southern Mentawai earthquake and tsunami an event which occurred at night, during a period of unsettled and rainy weather. The causative earthquake was also anomalous in that it only caused weak ground shaking and residents did not generally feel the need to spontaneously evacuate. Ultimately a tsunami with heights greater than 10 m tore through numerous coastal villages causing hundreds of deaths (Hill et al., 2012).

The early tsunami waves were also an important mitigating factor for the residents of the western coast of Tongatapu. Based on information from eyewitnesses, the first waves arrived largely without warning. These waves were large enough to inundate the western beaches and penetrate to the boundaries of the coastal properties, but they did not cause extensive inundation or damage. It was the effect of these waves that prompted the locals into action to evacuate guests and staff from the resorts. The loud booms following the arrival of the first waves as well as the atmospheric pressure fluctuations, i.e., “popping ears” then prompted people to accelerate their evacuation to higher ground. Due to the quick response following the initial surges, by the time the larger and more destructive waves arrived, the evacuation was well under way, allowing locals to get out of the area and reach high ground or elevated vantage points.

The eruption on 14th January 2022 (the day prior to the main eruption) also played a significant role in highlighting of the possibility of a tsunami being generated by a large eruption. The Tonga Meteorological service issued a Marine Tsunami Warning on the morning of the 14th which was circulated through radio and news outlets. While all tsunami warnings had been cancelled on the morning of the 15th, these warnings raised awareness in the coastal communities. Following the loud booms and observation of the ash column an urgent tsunami evacuation warning was issued and immediately played on AM radio at 425 UTC. This likely gave confirmation to those already evacuating and an additional nudge for others.

Another mitigating factor was the complete absence of international tourists in Tonga due to travel restrictions from the ongoing COVID-19 pandemic. Although resorts on the west coast were operating, they were at reduced capacity and catering only to domestic patrons. Despite this, the tsunami struck on a fine weekend, when domestic usage of beach and resort areas was highest.

The Government of Tonga and other international agencies can be credited with reducing tsunami casualties through their ongoing efforts of tsunami hazard mitigation through education and outreach. These efforts have been steadily increasing world-wide since the 2004 Indian Ocean tsunami and were significantly ramped up in Pacific Island nations following the 2009 Samoa-Tonga earthquake and tsunami. One initiative in particular, World Tsunami Awareness Day (WTAD), designated as the 5th of November each year by the UN General Assembly in 2015, may have been particularly beneficial to reducing casualties during the January 15th event. In Tonga, WTAD was commemorated through a series of educational and outreach initiatives just 2.5 months before the Hunga-Tonga event. Activities included art and poetry competitions and exhibitions in schools, discussions on Tongan radio stations, prayers and reminders during church services on the Sunday prior to WTAD, and a series of activities on the day itself –although this event was held a week later due to a COVID lockdown that was in effect on November 5th.

## Conclusion

On January 15, 2022, the eruption of the Hunga volcano generated a series of massive tsunamis causing large-scale destruction along the western shores of several Tongan islands. The tsunamis were likely generated by a combination of mechanisms including evacuation of water by explosive eruptions and atmospheric pressure waves radiating out from the volcanic explosion. Other volcanic processes, such as small-scale flank collapse, radial pyroclastic density currents entering the water, and caldera collapse may have contributed, but the timing and magnitude of these is still being evaluated. These multiple sources combined to produce a highly complex tsunami with catastrophic effects in the near field, as well as unusually persistent and damaging effects at distant locations around the Pacific and Atlantic Oceans. This was an unprecedented event in the written history of the Pacific basin; however, the large number of submarine and island volcanoes present around the ‘Ring of Fire’ suggest it was not a unique occurrence. Its closest historical analogue was the 1883 eruption of Krakatau in Indonesia, which caused a similarly large and globally observed tsunami (Matoza et al., 2022), albeit with a much higher death toll in the near source region. Indeed, the extraordinarily low number of casualties given the extreme devastation caused by this tsunami is a marvel in itself.

On Tongatapu, the survey recorded a peak tsunami height of approximately 19 m on the western coast of the Hihifo Peninsula near Liku’alofa. Around Tongatapu, tsunami heights were generally in excess of 15 m along the west coast, 2–4 m on the north coast, 10–15 m on the south coast and ~ 7 m on the east coast. On the west coast of ‘Eua tsunami heights were of the order of 5–10 m while the Nomuka and southern Ha’apai Islands, tsunami heights ranged from 5 to 20 m with ~ 20 m runup on the south shore of Tofua. The largest measured tsunami height from these surveys was tsunami runup along a cliff face at Nomuka Iki island where a height of 20.5 m was recorded amongst several flow marks in excess of 13 m. Inundation extents were in excess of 900 m where the tsunami surge crossed the Hihifo Peninsula but were more generally of the order of 200 m in the west and 100–200 m in Nuku’alofa. Shorter inundation distances were measured along the steep coral cliffs of the southern Tongatapu coast.

The tsunami was recorded on two tidal stations in Nuku’alofa. While the primary station failed during the tsunami, a full record of the tsunami was captured on a second station located 2 km to the west. This gauge recorded a peak tsunami height of 1.2 m at 0446 UTC (5:46 PM local time). The gauge also captured the signal of tsunami waves that were generated prior to the large explosion at 0415 UTC. These early waves were experienced by witnesses on the west coast of Tongatapu as a series of 3–4 surges that inundated over the beach and into coastal properties and served as a serendipitous early warning for people to evacuate the area immediately. A second series of explosions at ~ 0426–0427 UTC generated sonic booms, and likely additional waves. However, the largest surge that likely crossed the Hihifo Peninsula after ~ 0500 UTC is currently impossible to pin down to an obvious volcanic or tectonic source. Fortunately, nearly everyone in the area evacuated the beach front prior to the arrival of this largest surge, which caused complete destruction of the numerous beach resorts along the coast. Efforts to model the event, while generally accurate in terms of measured maxima and comparisons to available water level records are as yet unable to reproduce the necessary timing of the most destructive surge on the west coast of Tongatapu.

This event should serve as a ‘teachable moment’ for the hazard presented by volcanically generated tsunami. However, concentration on volcanic sources tsunami should not be at the expense of continued vigilance against tsunami generated from the Tonga Trench Subduction Zone to the east. Given the relative frequency of tsunamigenic events in the region, the Tonga Trench presents a much greater hazard relative to volcanic sources (Borrero et al., 2021). In contrast to this event, tsunami generated on the Tonga Trench will cause impacts that are more severe on the eastern and northern coasts of Tongatapu and the eastern coasts of the islands to the north. As such tsunami hazard mitigation efforts should be reinforced in these areas and use the lessons learned from the recent event to educate residents of the likely effects if a large-scale tectonic tsunami were to occur.

## Supplementary Information

Below is the link to the electronic supplementary material.

Supplementary Figure S1: Atmospheric pressure record from the Kanokupolu weather station. Data was recorded at 10 minute intervals and uploaded hourly on the hour. The final transmission was made at 1800 local time (0500 UTC) and clearly shows the arrival of the anomalous pressure drop caused by the eruption. (PNG 168 KB)

Supplementary Table S1 (DOCX 18 KB)

SupplementaryTable S2 (DOCX 37 KB)

Supplementary Video 1: Shot by crew member of Ngahau Siliva of tsunami waves shoaling. The video was shot at 0428 UTC (17:28 local) on January 15, 2022 from the location indicated in Fig. 1. The view is to the west-southwest (MOV 53968 KB)

Supplementary Video 2a: Recording of tsunami induced currents in Nuku’alofa Harbour on the afternoon of January 14th 2022 (MOV 58126 KB)

Supplementary Video 2b: Recording of tsunami induced currents in Nuku’alofa Harbour on the afternoon of January 14th 2022 (MOV 63505 KB)

Supplementary Video 3: Widely circulated and recovered from social media. The video shows the group of young men returning to the road from the beach. The extent of the volcanic mushroom cloud is evident. A first tsunami surge is seen coming from the north and sweeping southward causing the group to scramble to high ground. After a pause, the video continues now being shot from inside a vehicle and looking northward. Another tsunami surge comes from the north, this time inundating up the street where they were parked forcing them to hastily reverse as the tsunami advances knocking down a fence. The toppling of the fence can also be seen in Video 4 described below (MP4 16354 KB)

Supplementary Video 4a: Shot from further inland on the same street as Video 2. The first frame shows a car with its hatchback open. This is the car containing the people recording Video 2 described above. The tsunami surge can be seen advancing ad toppling the fence. A grey minivan is seen entering the frame from the left and turning right, away from the ocean. This car is followed by a white SUV which contains Australian High Commissioner Rachael Moore and her family. Ms. Moore took the photos in Fig.13 (a,b,c). At 0:13 seconds a loud report, similar to a gunshot or a thunder crack can be heard. This is one of the explosions emanating from the Hunga volcano (MP4 2122 KB)

Supplementary Video 4b: An edited video that combines Videos 3 and Video 4a. The video goes to split screen at the point where the two video begin to overlap. There is a pause at 00:29 to show that the two videos are in sync and again at 0:37 when Ms. Moore’s car is seen leaving the resort (MP4 110474 KB)

Supplementary Video 5: A video shot from Mr. Branko Sugar’s boat located 7.2 km NE of Nuku’alofa. The video starts at 04:25:36 UTC (17:25:36 local time) on January 15th. Booms can be heard at 0:10-0:13 in the video followed by some rumbling. Stronger/louder booms can then be heard at 1:08-1:09. At 1:23, someone says, “Look at the waves, look at the waves” and a faint bit of white water can be seen on the horizon near the right hand side of the frame (MOV 29432 KB)

## Data Availability

All data generated during this study are included in this published article and its supplementary information files. Tide gauge data plotted in the article are available from the corresponding author on reasonable request. Detailed tsunami runup data and individual measured transects are avaialble via the International Tsunami Information Center website.
