# Supplementary figures and images for: Tsunami Runup and Inundation in Tonga from the January 2022 Eruption of Hunga Volcano
Source: Pure Appl Geophys. 2022 Dec 28;180(1):1–22. doi: 10.1007/s00024-022-03215-5 (PMC9795157; doi:10.1007/s00024-022-03215-5)

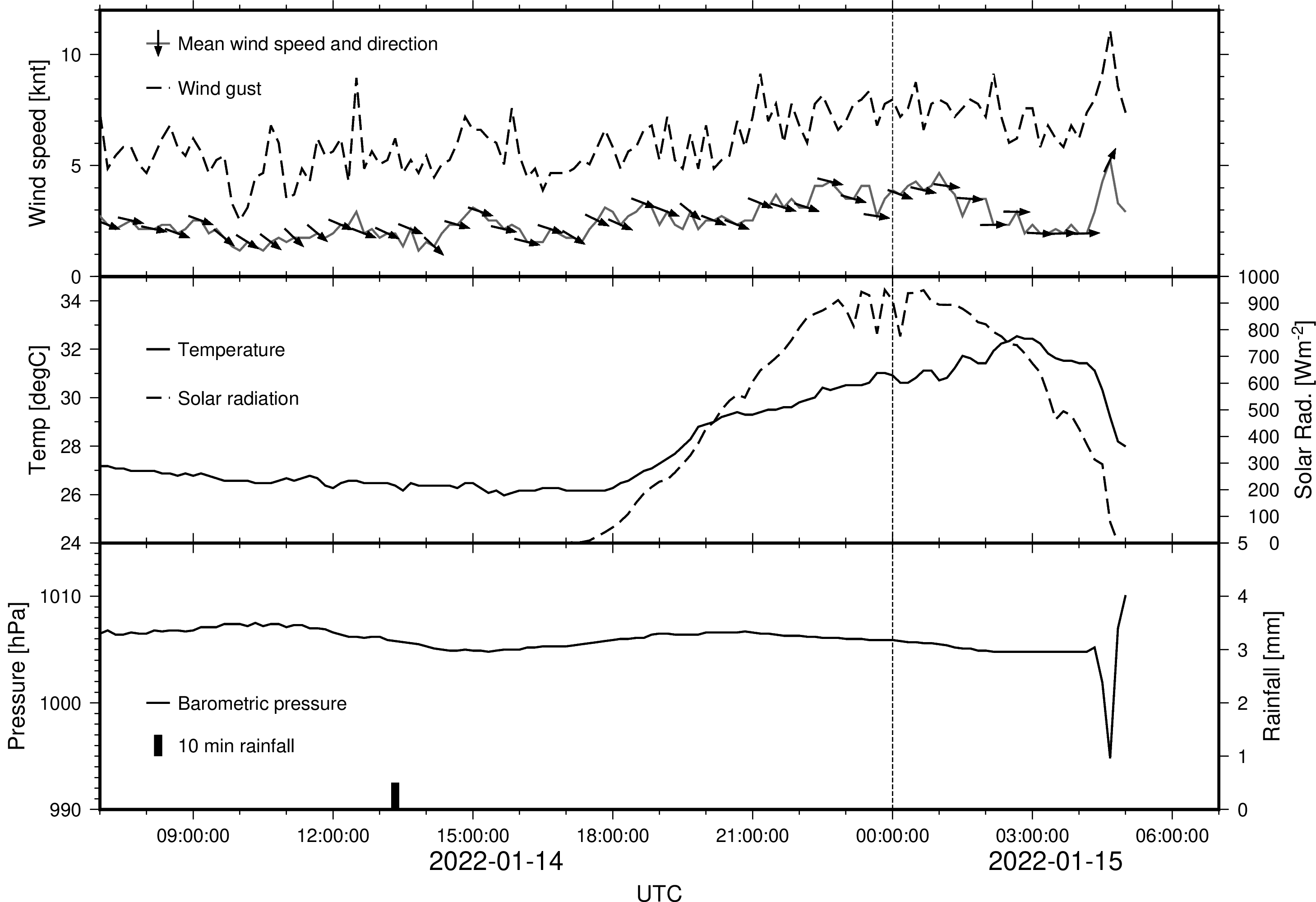

Supplement: Supplementary file 1 — Supplementary Figure S1: Atmospheric pressure record from the Kanokupolu weather station. Data was recorded at 10 minute intervals and uploaded hourly on the hour. The final transmission was made at 1800 local time (0500 UTC) and clearly shows the arrival of the anomalous pressure drop caused by the eruption. (PNG 168 KB) [file 24_2022_3215_MOESM1_ESM.png]
