# Supplementary material for: Tsunami Runup and Inundation in Tonga from the January 2022 Eruption of Hunga Volcano
Source: Pure Appl Geophys. 2022 Dec 28;180(1):1–22. doi: 10.1007/s00024-022-03215-5 (PMC9795157; doi:10.1007/s00024-022-03215-5)
Supplement: Supplementary file 2 — Supplementary Table S1 (DOCX 18 KB) [file 24_2022_3215_MOESM2_ESM.docx]

**Table S1** Some noteworthy and/or significant historical tsunami events affecting the Kingdom of Tonga. Source Validity: 4 = ‘definite’, 3 = ‘likely’, 2 = ‘questionable’, 1 = ‘doubtful’. Type: Eq. -= Earthquake, Vol. = Volcano, ?? = unknown (NGDC/WDC 2022).

| **Year** | **Date** | **Validity** | **Source Type** | **Earthquake Magnitude** | **Source Location** | **Source Latitude** | **Source Longitude** | **Max Water Height (m)** | **Measurement Type/Note** |
| --- | --- | --- | --- | --- | --- | --- | --- | --- | --- |
| 1853 | 24-Dec | 1 | Eq |  | Tonga Trench | -21.167 | -175.167 | inundation | Severe earthquake felt on Tongatapu. Anecdotal account from months after the event. Reported large scale subsidence on NE Tongatapu leading to >3 km of inundation from flooding. Western shore of island reportedly uplifted by ~1m and a new island formed offshore. Assumed to have been formed on the same night (Christmas Eve, 1853) when the ocean inundated Hihifo Peninsula (Western Tongatapu), however this may not be true. |
| 1865 | 18-Nov | 4 | Eq | 8.0 | Tonga Trench | -19.500 | -173.500 |  | Strong earthquake felt throughout Tonga. Account from ship caption of being run aground on Tau reef (near Nuku'alofa) then being re-floated by a series of tidal waves and pushed on to the reef flat. |
| 1877 | 10-Oct | 4 | Eq | 8.3 | Iquique, Chile | -21.500 | -70.500 | 3.60 | Vague newspaper account from Australia reported that 'the tidal wave at Tonga rose 12 feet. The beach is strewn with millions of fish.' Possibly erroneous or exaggerated. |
| 1889 | 8-Mar | 2 | ?? | - | Unknown | Unknown | Unknown | 8 to 11 | Unknown tsunami source, significant damage to coastal forest, large boulders moved, graveyard disturbed, effects on Lifuka and Foa Islands, Ha'apai group. Possibly cyclone storm surge. |
| 1892 | 30-Aug | 3 | Vol | - | Tofua? | -19.75 | -175.07 | ~1m | Withdrawals of water from harbour in Lifuka, boats left dry. Water returned but not to damaging height. |
| 1907 | July ?? | 2 | Vol | - | 50 km SW Tongatapu |  |  |  | ‘Tidal wave' reported along with pumice rafts. |
| 1917 | 26-Jun | 4 | Eq | 8.3 | Samoa | -15.500 | -173.000 | 2.74 | Earthquake between Tonga and Samoa, Tonga effects seen at Niuatoputapu. |
| 1919 | 30-Apr | 4 | Eq | 8.1 | Tonga Trench | -18.352 | -172.515 | 2.5 | Earthquake felt strongly in Ha'apai, small tsunami ~30 minutes later. |
| 1928 | 18-May | 2 | Eq | - | Tonga Trench | -19.830 | -174.360 |  | Earthquake felt on Lifuka. 'A tidal wave appeared, and pumice cast upon the shore.’ |
| 2009 | 29-Sep | 4 | Eq | 8.1 | Samoa/Tonga | -15.489 | -172.095 | ~20 m | Large earthquake along northern Tonga Trench SW of Samoa. Runup of up to ~20 m on Niuatoputapu with 7 deaths there (183 deaths in Samoa/Am. Samoa) Recorded at 0.15 m on Nuku'alofa tide gauge. 0.05 m on DART station. |
| 2011 | 11-Mar | 4 | Eq | 9.1 | Tohoku, Japan | 38.297 | 142.372 | 0.60 | Very large earthquake in Japan. Measured on tide gauge. |
| 2022 | 13-Jan | 4 | Vol | - | HTHH | -20.536 | -175.382 | 0.15 | Precursor eruption on Hunga Tonga-Hunga Ha'apai (HTHH) Volcano |
| 2022 | 14-Jan | 4 | Vol | - | HTHH | -20.536 | -175.382 | ~20 m | Large eruption of HTHH volcano. |
