# Supplementary material for: Tsunami Runup and Inundation in Tonga from the January 2022 Eruption of Hunga Volcano
Source: Pure Appl Geophys. 2022 Dec 28;180(1):1–22. doi: 10.1007/s00024-022-03215-5 (PMC9795157; doi:10.1007/s00024-022-03215-5)
Supplement: Supplementary file 3 — SupplementaryTable S2 (DOCX 37 KB) [file 24_2022_3215_MOESM3_ESM.docx]

**Table S2a**: Tsunami runup, maximum tsunami heights and inundation distances on Tongatapu.

| **Site** | **Long.** | **Lat.** | **Runup**  **(m)** | **Max**  **Elev.**  **(m)** | **Inund.**  **Dist.**  **(m)** | **Comment** |
| --- | --- | --- | --- | --- | --- | --- |
| ***Tongatapu*** |  |  |  |  |  |  |
| Atata 1 | -175.25458 | -21.05021 |  | 6.1 | >134 | Crossed entire peninsula 134 m distance |
| Atata 2 | -175.25590 | -21.04887 | 6.6 | 8.2 | 39.0 |  |
| Atata 3 | -175.25733 | -21.04630 | 9.1 | 10.5 | 91.0 |  |
| Atata 4 | -175.25610 | -21.05333 |  | 8.5 | >120 | Crossed entire peninsula |
| Fafa 1 | -175.16006 | -21.08732 |  | 6.3 | >330 | Crossed entire island |
| Fafa 2 | -175.16013 | -21.08708 |  | 7.5 | >200 | Crossed entire island - wave reinforced from two directions |
| Ha'atafu 1 | -175.33282 | -21.06721 | 14.9 | 16.7 | 117.0 |  |
| Ha'atafu 2 | -175.33355 | -21.06851 | 13.1 | 15.5 | 132.0 |  |
| Otuhaka | -175.33987 | -21.07553 |  | 14.3 | 880.0 | Passed from east to west across peninsula |
| NIWA/Weather Station | -175.34132 | -21.07722 |  | 17.5 | 706.0 | Passed from east to west across peninsula |
| Likualofa | -175.34516 | -21.08161 |  | 18.7 | 1120.0 | passed from east to west across peninsula |
| Kolowai | -175.35609 | -21.09531 | 14.8 | 16.9 | 218.0 |  |
| Samaletane 1 | -175.35579 | -21.09608 | 14.4 | 18.4 | 179.0 |  |
| Samaletane 2 | -175.35549 | -21.09695 | 14.3 | 14.7 | 180.0 |  |
| Samaletane 3 | -175.35483 | -21.09863 | 13.0 | 14.7 | 166.0 |  |
| Utukehe 1 | -175.34799 | -21.12366 | 16.7 | 16.7 | 253.0 |  |
| Utukehe 2 | -175.34774 | -21.12456 | 16.6 | 12.8 | 230.0 |  |
| Kala'au 1 | -175.34502 | -21.13716 | 12.7 | 16.3 | 216.0 |  |
| Kala'au 2 | -175.34537 | -21.13576 | 11.9 | 14.7 | 195.0 |  |
| Fanga Piliki 1 | -175.24876 | -21.19962 | 10.3 | 12.2 | 71.0 |  |
| Fanga Piliki 2 | -175.24778 | -21.19983 | 14.7 | 15.9 | 83.0 |  |
| Fanga Piliki 3 | -175.24637 | -21.19997 | 11.3 | 15.7 | 80.0 |  |
| Keleti 1 | -175.23484 | -21.20315 | 11.6 | 12.0 | 47.0 |  |
| Keleti 2 | -175.23415 | -21.20294 | 11.8 | 11.8 | 57.0 |  |
| Keleti 3 | -175.23313 | -21.20309 | 11.5 | 12.2 | 44.0 |  |
| Keleti 4 | -175.23266 | -21.20361 | 9.8 | 11.4 | 70.0 |  |
| Hufangalupe 1 | -175.19048 | -21.23039 | 15.6 | 15.6 | 40.0 |  |
| Hufangalupe 2 | -175.19037 | -21.22933 |  | 12.9 | 13.0 |  |
| Hufangalupe 3 | -175.19032 | -21.23013 | 14.6 | 14.6 | 14.0 |  |
| Ahononou 1 | -175.17081 | -21.25691 | 14.6 | 14.6 | 86.0 |  |
| Ahononou 2 | -175.17103 | -21.25663 | 12.2 | 12.2 | 106.0 |  |
| Ahononou 3 | -175.16877 | -21.25793 | 13.6 | 13.8 | 67.0 |  |
| Katea 1 | -175.12662 | -21.27294 | 12.7 | 12.8 | 13.0 |  |
| Katea 2 | -175.12574 | -21.27276 | 10.7 | 10.7 | 12.0 |  |
| Halaika 1 | -175.09944 | -21.23269 | 6.8 | 6.8 | 93.0 |  |
| Halaika 2 | -175.09886 | -21.23504 | 6.2 | 6.2 | 75.0 |  |
| Emeline 1 | -175.05101 | -21.16539 | 6.4 | 6.4 | 24.0 |  |
| Emeline 2 | -175.05126 | -21.16546 | 5.6 | 5.6 | 24.0 |  |
| Emeline 3 | -175.04981 | -21.16502 | 6.4 | 6.4 | 25.0 |  |
| Fanga  Taupo'ou 1 | -175.03595 | -21.14310 | 4.5 | 4.5 | 22.0 |  |
| Fanga  Taupo'ou 2 | -175.03556 | -21.14348 | 3.1 | 3.1 | 14.0 |  |
| Kolonga 1 | -175.07281 | -21.12499 | 2.3 | 2.3 | 13.0 |  |
| Kolonga 2 | -175.07277 | -21.12502 | 3.2 | 3.2 | 18.0 |  |
| Kolonga 3 | -175.07314 | -21.12481 | 2.4 | 2.4 | 14.0 |  |
| Manuka | -175.09645 | -21.12041 | 1.0 | 2.8 | 150.0 |  |
| Talafo'ou  lagoon | -175.12118 | -21.13663 | 1.2 | 1.2 | 22.0 | eyewitness |
| Lapaha  lagoon | -175.11855 | -21.17528 | 0.9 | 0.9 | 12.0 | eyewitness |
| Ha'ateiho  lagoon | -175.22920 | -21.17554 | 0.4 | 0.2 | 1.0 | eyewitness |
| Halaleva  lagoon | -175.18917 | -21.15344 | 0.7 | 0.7 | 3.0 | eyewitness |
| Patangata 1 | -175.15416 | -21.13865 | 0.4 | 2.5 | 158.0 | eyewitness |
| Patangata 2 | -175.15874 | -21.14032 | 1.2 | 3.6 | 102.0 | eyewitness |
| Popua - North 1 | -175.16267 | -21.14112 | 1.1 | 2.8 | 230.0 |  |
| Popua - North 2 | -175.16129 | -21.14095 | 2.4 | 3.0 | 63.0 |  |
| Nukualofa  Seaview | -175.20365 | -21.12926 | 3.4 | 3.5 | 93.0 | eyewitness |
| Nukualofa  Convention | -175.19570 | -21.13377 | 1.8 | 2.5 | 112.0 | eyewitness |
| Nukualofa  Villa/Bakery | -175.19025 | -21.13640 | 1.2 | 2.8 | 343.0 | eyewitness |
| Nukualofa  Wharf | -175.18336 | -21.13879 | 2.2 | 2.3 | 138.0 | eyewitness |
| Nukualofa  East Vuna | -175.17372 | -21.14062 | 1.9 | 2.8 | 244.0 |  |
| Sopu 1 - West | -175.22880 | -21.11935 | 2.0 | 2.6 | 63.0 | eyewitness |
| Sopu 2 | -175.22294 | -21.12358 | 1.4 | 3.5 | 137.0 | eyewitness |
| Sopu 3 | -175.21522 | -21.12551 | 1.6 | 4.2 | 256.0 | eyewitness |
| Sopu  Captain Cook | -175.20963 | -21.12705 | 2.0 | 3.9 | 168.0 | eyewitness |
| Nukunuku | -175.29567 | -21.12463 | 1.2 | 0.3 | 3.0 |  |
| Te'ekiu | -175.31586 | -21.11967 | 2.2 | 0.6 | 29.0 | eyewitness |

**Table S2b**: Tsunami runup, maximum tsunami heights and inundation distances **i**n the northern islands.

| **Site** | **Long.** | **Lat.** | **Runup**  **(m)** | **Max**  **Elev.**  **(m)** | **Inund.**  **Dist.**  **(m)** | **Comment** |
| --- | --- | --- | --- | --- | --- | --- |
| ***Ha'apai*** |  |  |  |  |  |  |
| Nomuka iki cliff | -174.80653 | -20.28584 | 20.5 | 20.5 |  | Flow heights measured along cliff (variable wave edge) 13.5-20.5 |
| Nomuka iki 1 | -174.80712 | -20.28382 |  | 12.6 | 565.0 | Flowed across the low forested part of the island (stripped forest) |
| Nomuka iki 2 | -174.80629 | -20.28358 | 17.5 | 17.5 | 115.0 |  |
| Tonumea 1 | -174.76109 | -20.46245 | 7.1 | 12.9 | 108.0 |  |
| Tonumea 2 | -174.76042 | -20.46180 |  | 14.6 | >193 | Flowed across peninsula and two waves met |
| Tonumea 3 | -174.76255 | -20.46085 | 12.2 | 12.2 | 42.0 |  |
| Tonumea 4 | -174.76226 | -20.46126 | 10.3 | 10.3 | 45.0 |  |
| Nomuka 1 | -174.79465 | -20.26324 | 6.4 | 10.0 | 125.0 |  |
| Nomuka 2 | -174.79542 | -20.26281 | 5.0 | 7.3 | 141.0 |  |
| Nomuka 3 | -174.79658 | -20.26212 |  | 5.4 | 163.0 | Passed through the village into the lake behind |
| Nomuka 4 | -174.79785 | -20.26051 | 5.9 | 6.6 | 75.0 |  |
| Mango 1 | -174.71530 | -20.32444 | 7.5 | 9.1 | 306.0 |  |
| Mango 2 | -174.71682 | -20.32512 | 7.4 | 9.1 | 380.0 |  |
| Fonoi 1 | -174.63166 | -20.28020 | 4.9 | 5.4 | 98.0 |  |
| Fonoi 2 | -174.63192 | -20.28030 | 2.6 | 4.0 | 124.0 |  |
| Tungua 1 | -174.75969 | -20.01728 | 1.0 | 2.6 | 181.0 |  |
| Tungua 2 | -174.75919 | -20.01580 | 0.7 | 4.1 | 185.0 |  |
| Tofua East 1 | -175.02990 | -19.75069 | 14.0 | 14.0 | 30.0 | Measured via pole and pace |
| Tofua East 2 | -175.02992 | -19.75031 | 16.0 | 16.0 | 30.0 | Measured via pole and pace |
| Tofua SW 1 | -175.08223 | -19.77932 | 20.6 | 20.6 | 54.5 | Measured via pole and pace |
| Tofua SW 2 | -175.08223 | -19.77932 | 22.0 | 22.0 | 35.0 | From GPS |

**Table S2c**: Tsunami runup, maximum tsunami heights and inundation distances on Eua.

| **Site** | **Long.** | **Lat.** | **Runup**  **(m)** | **Max**  **Elev.**  **(m)** | **Inund.**  **Dist.**  **(m)** | **Comment** |
| --- | --- | --- | --- | --- | --- | --- |
| **Eua** |  |  |  |  |  |  |
| Blue Water Retreat 1 | -174.96908 | -21.36090 | 5.3 | 5.4 | 89.4 |  |
| Blue Water Retreat 2 | -174.96936 | -21.36151 | 4.1 | 5.6 | 76.2 |  |
| Ohonua Trench | -174.95495 | -21.33871 | 15.8 | 16.1 | 342.8 | end of transect, but not end of inundation. Couldn't continue along path |
| Ha'atu’a 1 | -174.97163 | -21.39650 | 7.5 | 7.5 | 82.2 | log pile at runup |
| Ha'atu’a 2 | -174.97247 | -21.39468 | 7.5 | 8.0 | 86.3 | debris pile |
| Ohonua 2 | -174.95780 | -21.34080 | 3.6 | 6.8 | 191.1 |  |
| Ohonua 1 -1 | -174.95999 | -21.34422 | 4.9 | 8.4 | 195.8 |  |
| Ohonua 1-2 | -174.95787 | -21.34473 | 5.5 | 8.1 | 237.2 |  |
| Ta'anga 1 | -174.95214 | -21.33521 | 7.0 | 7.0 | 133.5 |  |
| Ta'anga 2 | -174.95068 | -21.33369 | 5.0 | 5.3 | 102.5 |  |
| Tufuvai 1 | -174.96470 | -21.35079 | 9.3 | 9.3 | 149.8 |  |
| Tufuvai 2 | -174.96448 | -21.34999 | 7.1 | 7.8 | 177.7 | debris pile on huge coral terrace |
| Ohonua 3 | -174.95709 | -21.33970 | 3.0 | 5.8 | 198.7 |  |
